# Quantitatively probing the magnetic behavior of individual nanoparticles by an AC field-modulated magnetic force microscopy

**DOI:** 10.1038/srep22467

**Published:** 2016-03-02

**Authors:** Xiang Li, Wei Lu, Yiming Song, Yuxin Wang, Aiying Chen, Biao Yan, Satoru Yoshimura, Hitoshi Saito

**Affiliations:** 1 School of Materials Science and Engineering, University of Shanghai for Science and Technology, Shanghai 200093, China; 2 School of Materials Science and Engineering, Tongji University, Shanghai 201804, China; 3 Research Center for Engineering Science, Graduate School of Engineering & Resource Science, Akita University, Akita 010-8502, Japan

## Abstract

Despite decades of advances in magnetic imaging, obtaining direct, quantitative information with nanometer scale spatial resolution remains an outstanding challenge. Current approaches, for example, Hall micromagnetometer and nitrogen-vacancy magnetometer, are limited by highly complex experimental apparatus and a dedicated sample preparation process. Here we present a new AC field-modulated magnetic force microscopy (MFM) and report the local and quantitative measurements of the magnetic information of individual magnetic nanoparticles (MNPs), which is one of the most iconic objects of nanomagnetism. This technique provides simultaneously a direct visualization of the magnetization process of the individual MNPs, with spatial resolution and magnetic sensitivity of about 4.8 nm and 1.85 × 10^−20^ A m^2^, respectively, enabling us to separately estimate the distributions of the dipolar fields and the local switching fields of individual MNPs. Moreover, we demonstrate that quantitative magnetization moment of individual MNPs can be routinely obtained using MFM signals. Therefore, it underscores the power of the AC field-modulated MFM for biological and biomedical applications of MNPs and opens up the possibility for directly and quantitatively probing the weak magnetic stray fields from nanoscale magnetic systems with superior spatial resolution.

Over the past few decades, the magnetic properties of iron oxide nanoparticles have been widely explored mainly due to their potential applications in various fields, including storage media, environmental remediation, and biomedicine^1^,^2^,^3^. The most common magnetic material in nature is magnetite (Fe3O4). The size of the magnetic particle is a critical factor that affects its magnetic properties. Depending on their magnetic properties, magnetite particles between 35 to 80 nm in diameter are likely to be single-domain magnets^4^. Magnetite particles with diameters less than 35 nm are believed to be superparamagnetic because they do not have a sufficient volume to ensure a stable magnetic moment, as thermal energy can reverse its moment. Superparamagnetic Magnetite nanoparticles (SPNs) can be readily functionalized for specific binding to a wide variety of molecules^5^,^6^,^7^, and are thus particularly useful for biological detection and imaging applications^8^,^9^.

For research purposes as well as for quality control, a precise characterization of the magnetic properties of individual magnetic nanoparticles is essential. The fundamental prerequisite for such characterization is to have probing technique at hands. This in its own is a real challenge. In the case of magnetic systems, although a remarkable number of magnetic microscopy techniques have been developed over the last decades, imaging magnetism at the nanoscale remains a challenging task because it requires a combination of high spatial resolution and sensitivity^10^. Up to now, only a few methods have been proven to have the sensitivity for detecting the magnetic behavior of single nanoparticles below 35 nm. Recently, insights into the magnetic properties of individual and isolated particles below 35 nm were obtained with the help of anomalous Hall-effect (AHE)^11^, superconducting quantum interference device (SQUID)^12^,^13^, spin-polarized scanning tunneling microscopy^14^, transmission X-ray microscopy^15^ and nitrogen-vacancy magnetometer^16^. However, these techniques require highly complex experimental apparatus and a dedicated sample preparation so that the particles can reach and escape from the tested region without perturbations. In addition, the most sensitive of these magnetometers generally require low temperatures for operation, but the ability to measure under ambient conditions (standard temperature and pressure) is critical for many imaging applications, particularly in biological systems^17^.

To observe magnetic samples in their real, unprepared state, a more suited approach consists in mapping the magnetic stray field generated outside the sample. Furthermore, the spatial resolution is then limited both by the probe size and its distance to the sample. Among many stray-field microscopy techniques^18^, magnetic force microscopy (MFM) has become ubiquitous, as it provides a spatial resolution <30 nm and operates under ambient conditions without any specific sample preparation, enable to provide microscopic information about the magnetic behavior of SPNs^19^,^20^. It appears straightforward to use methods of high-resolution MFM (i.e., to employ sharp MFM tips) to measure changes in the magnetic field caused by nanoparticle magnetization. However, as this magnetization is usually very small (~10^−19^ A m^2^ for a 10 nm-sized spherical iron oxide nanoparticle at saturation), such measurements require very sensitive methods. The ability of MFM to detect superparamagnetic and low-coercivity magnetic nanoparticles (MNPs) and the interpretation of the resulting MFM images are subjects of ongoing research. Agarwal^21^ reported the use of MFM to detect probe–sample interactions from SPNs which consisting of an iron oxide core (diameter 10 nm) surrounded by a dextran shell (giving a final diameter of 30 to50 nm) *in vitro* in ambient atmospheric conditions. Sievers^19^ described the quantitative measurement of the magnetization of individual MNPs (with diameter of about 19 nm) using MFM and a resolution of the magnetic moment in the order of 10^−18^ A·m^2^ under ambient conditions was demonstrated. Recently, very high sensitivity has been demonstrated by Dietz^22^, who detected the magnetic signal from single ferritin molecules in liquid using a multifrequency method based on harmonic distortion analysis, yet with difficult data interpretation. Using a Ferritin-based new MFM probe, magnetic interactions of approximately 10 nm sized magnetic nanoparticles were detected using MFM by Park^20^. However, the capability of MFM to detect a signal from MNPs has not been fully explored. A systematic and quantitative study of the applicability of MFM for characterizing MNPs in ambient condition with spatial resolution better than 10 nm is still lacking.

Often, magnetic forces are measured indirectly by recording a shift in MFM cantilever resonance frequency. However, for small nanoparticles this shift is very small and (for small distances to the nanoparticle) often distorted by a topological crosstalk. To circumvent this problem, in this work, we introduce an AC field-modulated MFM technique, which uses the frequency modulation of a cantilever oscillation by applying ac magnetic field to a mechanically oscillated tip, to locally characterize the magnetization behavior of individual MNPs with diameter of about 10 nm in atmosphere, as well as the nanoscale magnetic domains. To our knowledge, studies using MFM to investigate the magnetic information of individual MPNs with spatial resolution of 5 nm have rarely been reported. Compared with conventional MFM, the AC field-modulated MFM technique enables us to quantitatively map the stray field above individual MNPs with high spatial resolution, revealing in three dimensions the full structure of the magnetic field distribution, including the detection of more magnetic signals used for the analysis of nanoscale magnetic features, such as alternating field signals (standard MFM can only detect static field signals) and polarity of magnetic pole of the samples. Furthermore, we demonstrate simultaneously a direct visualization of the magnetization process of the individual MNPs. This work thus underscores the power of the AC field-modulated MFM for the applications in MNPs and opens new avenues for fundamental studies in nanoscale magnetic systems and nanomagnetism.

Figure 1 shows the schematic diagram of the AC field-modulated MFM system. The system was based on a conventional JSPM-5400(JEOL Ltd.) scanning probe microscope. In the system, a homogeneous AC magnetic field (with a frequency of 100 Hz and an amplitude of 0 ~ 7.2Oe) which can periodically change the magnetic domain states of the MNPs and induce an alternating force between the MFM tip and magnetic sample, is generated by a soft magnetic ferrite core, which is driven by an ac voltage with a frequency of ω_m_ from a signal generator. The cantilever is oscillated by a piezoelectric element with an oscillation frequency ω_c_ while the AC magnetic field modulates the effective spring constant of the cantilever. The cantilever deflections are sensed by laser beam deflection. The MFM signal is demodulated using a PLL circuit (easyPLL, Nanosurf ^®^). The signal out of PLL is fed into a lock-in amplifier and the ac voltage applied to the magnetic nanoparticles is used as a reference signal. In-phase amplitude signals of the lock-in amplifier are obtained by adding an extra phase so that the out-of-phase signals become zero. In this case, the phase shift arising from the electronics can be compensated.

The principle of AC field-modulated MFM is based on the frequency modulation (FM) of the cantilever resonance^23^. Assuming a hard magnetic MFM tip is used for measurement and the magnetization direction of the tip is perpendicular to the sample surface, it can be considered that the magnetization direction of the MFM tip will not be changed or moved during measurement and the tip behaves as a monopole type tip. When a magnetic nanoparticle sample is driven by an alternating magnetic field with a frequency of ω_m_, the magnetic moment (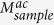) of the nanoparticle rotates periodically and it can be given by equation (1)


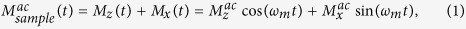


where 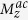 and 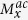 are the amplitudes of perpendicular and in-plane magnetization components, respectively.

Based on the frequency modulation of the cantilever resonance, the motion equation of the MFM tip can be given by


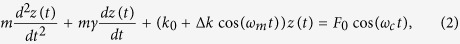


where *z* is the displacement of the tip in perpendicular direction, *m* the effective mass of the tip, γ the damping factor of the oscillation, *k*_0_ the intrinsic spring constant of the cantilever, 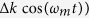 represents the periodic change of the effective spring constant of the cantilever due to the AC magnetic field and 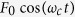 donates the oscillating force driven by a piezoelectric element with a frequency of ω_c_. When a MFM tip behaves as a monopole type tip, Δk can be given by


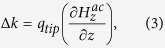


where 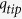 is the magnetic pole of the tip, 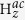 is a component of alternating magnetic field in a direction displacement of the tip in perpendicular direction. Therefore, the cantilever displacement can be given by


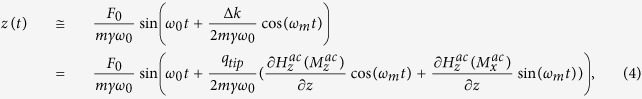


Hence, after the frequency modulation signal of the oscillation of the tip generated by the AC magnetic field is subjected to frequency demodulation, the demodulated signal is subjected to lock-in detection at the frequency ω_m_ of AC voltage signal using a lock-in amplifier with an output of the ac voltage as the reference signal. Thus, it is possible to perform the measurement of the magnetic field gradient 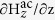
of the perpendicular magnetic field 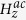 from the magnetic samples.

Here, the sign (upward and downward polarities) of 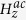 reflects the polarity (N and S poles) of the surface magnetic pole of the magnetic samples, and when the sign is reversed, the change of the effective spring constant can be given by


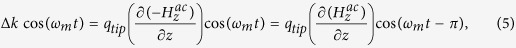


and the phase is changed by 180 degree. Thus, it is also possible to directly detect the sign of 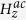 reflecting the polarity of the surface magnetic pole of the magnetic samples.

## Results

### Imaging the magnetic nanoparticles

The topographic, amplitude, and phase images were recorded using the AC field-modulated MFM with lift mode option, which allows to image relatively weak but long-range magnetic interactions, while minimizing the influence of the topography. In general, MNPs of less than 30 nm are superparamagnetic and can maintain their magnetization only in the presence of an external magnetic field at room temperature. In order to perform MFM experiments in an external magnetic field, a home-built soft ferrite core was mounted in the sample holder (refer to the above introduction). The ferrite core can generate a homogeneous alternating magnetic field at the sample surface with tunable amplitude by adjusting the amplitude of ac voltage from a signal generator. Therefore, field-dependent magnetic contrasts obtained in AC field-modulated MFM experiments can be exploited to get information about the magnetization behavior of individual magnetic nanoparticles.

In Fig. 2, we give an example of the typical AFM and MFM images of Fe_3_O_4_ nanoparticles. The topography (left), amplitude (middle) and phase (right) images were recorded without any external magnetic field applied. The topography image shows typical particle-like features, probably originates from Fe_3_O_4_ nanoparticles and gold nanoparticles. The MFM images, obtained by scanning the same area at a lift height of 10 nm above the surface of the sample, are related to the magnetic signals. However, no magnetic signals can be detected from the amplitude and phase images in Fig. 2. Further, MFM phase images (Fig. 3(b~f)) of Fe_3_O_4_ nanoparticles exhibited a strong contrast (dipole) in the presence of an externally applied magnetic field. These data confirmed that the external magnetic field was a prerequisite to detect SPNs for the MFM imaging and applied magnetic field is sufficient to induce a stable magnetic moment in SPNs at room temperature. In addition, it also indicates that signals in the lift mode originated only from the magnetic interaction between the MFM tip and magnetic nanoparticles.

Further experiments were performed to investigate the impact of external magnetic field. As the external field was varied, significant changes can be observed in the MFM images (Fig.3) while no change in the topographic images, confirming that the magnetic images are not appreciably influenced by the topography. As a consequence, when discussing the response of the nanoparticles as a function of external magnetic field, we shall restrict to the MFM images.

Figures 3 show the magnetization process of individual nanoparticles by applying a series of magnetic fields (a: 0 Oe, b: 2.2 Oe, c: 3.2 Oe, d: 5.1 Oe, e: 6.2Oe, f: 7.2 Oe). It can be found that, the magnetic contrast of the Fe_3_O_4_ magnetic nanoparticles changes gradually and uniformly with the field, which is due to the change of the average magnetization of the nanoparticles and thus modifies the average magnetic contrast. Each magnetic nanoparticle shows a dipolar response (bright and dark, which indicate an attractive and repulsive magnetic interaction between MFM tip and nanoparticles, respectively) after application of various external magnetic fields, providing a support for the single-domain structure in the nanoparticles. Similar MFM images with dipolar response have been observed in iron oxide SPNs^21^. This field-dependent magnetization of dipolar moment in the individual nanoparticles helps to confirm the presence of magnetic interaction between the MFM tip and the magnetic nanoparticle.

The overall variation can easily be separated from the localized changes of magnetic contrast observed in the regions containing the magnetic nanoparticles. Obviously, only several Fe_3_O_4_ magnetic nanoparticles can be detected at a small magnetic field of 2.2 Oe (fig. 3(b)). When a series of higher magnetic fields was applied, more magnetic nanoparticles were detected in the MFM images, indicating that more nanoparticles were magnetized successively with increasing magnetic field, as shown in Fig. 3(c–f).

Figure 4(a) reveals the fine magnetic domain structures of individual magnetic nanoparticles corresponding to the blue line area in fig. 3(f) with group of six single nanoparticles numbered as 1 to 6, together with MFM signal profiles across the center of the selected nanoparticles. The results imply that the six nanoparticles are in a single domain state with domain size of about 14 nm (estimated from line profile), which is a little larger than the result determined from TEM experiments (~10 nm). This is mainly resulted from the magnetic stray field at the boundary of MNPs. Fig. 4(b) shows the corresponding 3-Dimensional (3D) MFM image of indexed six nanoparticles and Fig. 4(c) is the 3D MFM image of particle 6. The boundaries between magnetic nanoparticles and nonmagnetic substrate are clearly distinguished, and the smooth transition from positive phase to negative phase in the dipolar moment of nanoparticles is also observed, from the 3D images. In addition, we can distinctly observe the force gradient and its distribution resulted from the interaction of magnetic nanoparticles and MFM tip.

The spatial resolution of MFM measurement in current study is estimated from the spectrum line profile of MFM phase signal. The resolution is determined as the full width at half maximum (FWHM) of peak signals. As shown in Fig. 4(a), the determined resolution is around 4.8 nm under the ambient condition with a lift height of 10 nm.

### Quantitative measurement of magnetization moment of individual MNPs

To confirm that our MFM experiments were able to detect the weak magnetic interaction between the MFM tip and MNPs, an analytical modeling of the MFM tip–sample interaction was performed. In the analytical approach, the nanoparticle is modeled as a single-domain magnetic particle of diameter d and magnetic moment ms. The tip magnet was modeled as a hollow shell of uniformly magnetized magnetic material with saturation magnetization Mp, outer shell radius R_1_ for the magnetic coating (typically 20 nm) and inner shell radius R_2_ (typically 10 nm as specified by tip supplier) for the silicon tip skeleton. The tip and the particle were separated by a distance s, which is related to the “lift-height” parameter and the amplitude of cantilever oscillations. It can be shown that, using the small cantilever oscillation amplitude approximation, the MFM signal (phase shift δφ) resulting from the dipolar tip–sample interaction can be given by^21^


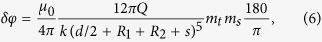


where k is the spring constant of MFM cantilever, Q is its quality factor, μ_0_ is the permeability of a vacuum, and m_t_ is the tip magnetic moment, given as:


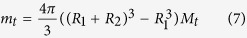


Here M_t_ (~1.2 × 10^6^ A/m) is the saturation magnetization of the FePt magnetic coating of the MFM tip. Using this equation m_t_ was estimated to be approximately 1.31 × 10^−16^ A m^2^.

To use Equation 1 to estimate the magnetic moment m_s_ of nanoparticles, the phase shift of magnetic nanoparticles needs to be determined from MFM phase image. As shown in Fig. 4, the MFM phase image and corresponding signal line profile of five individual MNPs are illustrated. The maximum phase shift of all nanoparticles was derived from the line profile. From the maximum phase shift 0.299 of particle 1, the absolute value of the magnetization moment is determined to be 2.76 × 10^−19^ A m^2^, using k = 42 N/m and experimental parameters given above. The magnetic moments of the other MNPs in Fig. 4 were determined accordingly, and the results are summarized in Table 1.

## Discussions

In this work, it is illustrated that AC field-modulated MFM technique enables local and quantitative measurements of the magnetic information of individual MNPs (10 nm in size), which is one of the most iconic objects of nanomagnetism, and therefore appears as a powerful tool for fundamental studies in nanomagnetism.

Results revealed that an external magnetic field of several Oe is sufficient to induce a stable magnetic dipole in MNPs at room temperature and the presence of an external magnetic field is essential to detect and distinguish an MFM signal from the MNPs. It is indicated that the magnetization behavior of individual MPNs can be probed by simply observing the magnetic domains under an applied field and we can separately estimate the distributions of the dipolar fields and the local switching fields of individual MNPs using the described AC field-modulated MFM technique. Moreover, the direct visualization of the field-dependent domain structure, allows the understanding of the magnetization reversal mechanism of individual MNPs. Indeed, the direct measurement of weak magnetic stray fields originated from MNPs with a nanoscale resolution under ambient conditions, may allow clearing some important issues, such as the nature of magnetic domain in nanoscale magnetic systems, detection of MNPs in immunolabeling and et al.

Based on the current work, the spatial resolution of the AC field-modulated MFM imaging of MNPs was determined to be around 4.8 nm under the ambient condition, which is much better than conventional MFM technique (~10 nm)^24^. In the AC field-modulated MFM, we use a lock-in amplifier, which enables us to detect smaller AC force signal than the conventional MFM. In addition, one advantage that we can get from the AC modulation technique is the discrimination of magnetic force from topographical forces. Therefore, we can approach the MFM tip much close to the sample and improve the spatial resolution of MFM. And another advantage is that a larger stray field can be generated from the magnetic nanoparticles in the AC magnetic field modulation technique, which gives higher MFM signal amplitude. The smaller minimal measurable force signal, higher signal amplitude, and better signal to noise ratio result in a better resolution in AC field-modulated MFM than the conventional one^25^.

We measured the phase shift of the MNPs in MFM signals to estimate and quantify the magnetization moment of individual MNPs. Analysis of the phase shifts in our MFM data suggests that the tip-sample interaction follows the interaction of single-domain magnetic particles with an MFM tip magnet, as defined in Equation (6) and also predicted by others^19^,^20^,^21^. Using this equation, the magnetic moment of MNPs analyzed in our MFM experiments was estimated. The magnetic moments m_s_ measured for individual nanoparticles in our study are closely consistent with previous experimental and calculated results of iron oxide SPNs^19^,^20^,^21^. In ref. 19, the m_s_ for individual 10 nm iron oxide SPNs was estimated to be 2.5 × 10^−19^ A m^2^ using data obtained from a superconducting quantum interference device (SQUID). The good agreement between these values supports the hypothesis that the phase shift originated from the magnetic interaction alone.

In current experiment, the value of magnetic moment to be reliably resolved is limited by the resolution of our MFM system which is based on a commercial scanning probe microscopy (JSPM-5400). The noise level estimated from MFM image is less than 0.02 degree. In this case, we may assume that phase shift larger than 0.02 degree can be reliably characterized. Therefore, the minimum resolvable magnetic moment is determined to be about 1.85 × 10^−20^ A m^2^, which corresponding to the magnetic moment of MNP with 4.2 nm in diameter (The magnetic moment of 4.2 nm MNP was estimated by considering the volume ratio between 10 nm MNP (2.5 × 10^−19^ A m^2^) in the literature and the above). The magnetic sensitivity (minimum resolvable magnetic moment) agrees well with the spatial resolution (4.8 nm) estimated above in our AC field-modulated MFM system. Smaller lift heights would mean higher magnetic sensitivity, however, and then the contribution of nonmagnetic interactions could gain importance. For common tip geometries a larger tip volume and a higher magnetization of the tip coating would lead to a higher net magnetic moment of the tip, thereby enhancing the sensitivity of the technique as long as the perpendicular alignment of the tip magnetization can be maintained.

In summary, present work marks a significant step forward in the investigation of MNPs. We expect that it will underscore the power of the MFM for biological and biomedical applications of magnetic nanoparticles, as well as broader applications in the study of nanomagnetism. The AC field-modulated MFM technique can be applied to investigate the microscopic magnetic domain structures in a variety of magnetic materials, such as nanoparticles, high density recording media, , patterned elements, as well as other magnetic features and nanostructures, which opens up the possibility for directly and quantitatively probing the weak magnetic stray fields from nanoscale magnetic systems with superior spatial resolution.

## Methods

Silicon wafers are used as substrates, and cut into pieces of approx. 10 × 20 mm^2^, which were ultrasonically cleaned with distilled and ion exchanged water before usage. For unspecific adsorption of nanoparticles on substrates, a Fe_3_O_4_ solution is prepared by solving 50 mg Fe_3_O_4_ powders in 100 ml distilled water. Then, only one half of the silicon slide is covered by a drop of Fe_3_O_4_ nanoparticle solution (concentration 0.5 mg/ml, adsorption time 12 minutes). Afterwards, the whole slide was dried naturally at 60 °C for 30 min in air. After drying, the nanoparticle-covered part of the surface is distinguishable from the uncoated one with the bare eye and redundant areas of the slide are removed from the sample by cutting. Finally, to fully immobilize the Fe_3_O_4_ nanoparticles on the slide and distinguish magnetic nanoparticles from nonmagnetic nanoparticles, 5 nm height gold particles were sputtered on the surface.

**Figure 1:**
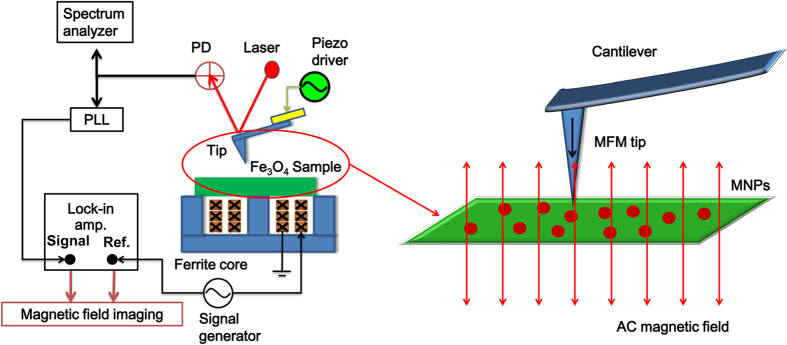
Schematic diagram of alternating magnetic field-assisted MFM system.

**Figure 2:**
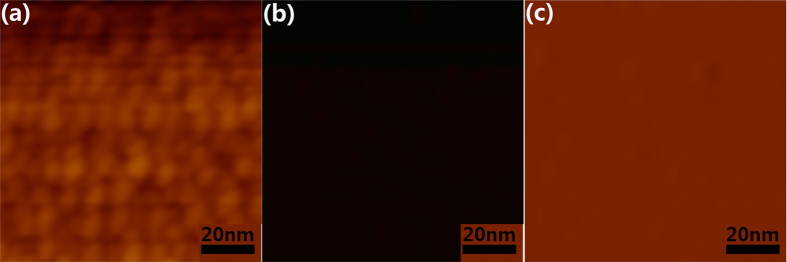
AFM and MFM images of Fe_3_O_4_ nanoparticles without any external magnetic field applied. (**a**) topography; (**b**) amplitude; and (**c**) phase images.

**Figure 3:**
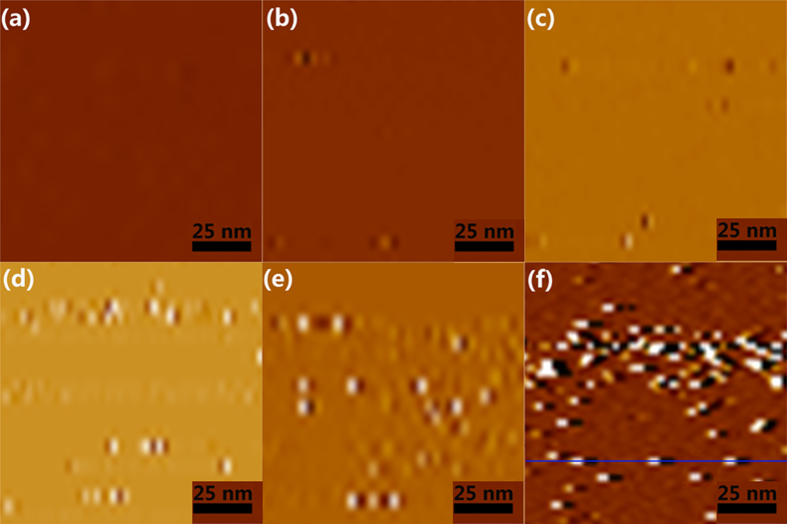
MFM images in the same area of sample Fe_3_O_4_ with an external applied magnetic field varied as follows: (**a**) 0 Oe , (**b**) 2.2 Oe, (**c**) 3.2 Oe, (**d**) 5.1 Oe, (**e**) 6.2Oe, (**f**) 7.2 Oe.

**Figure 4:**
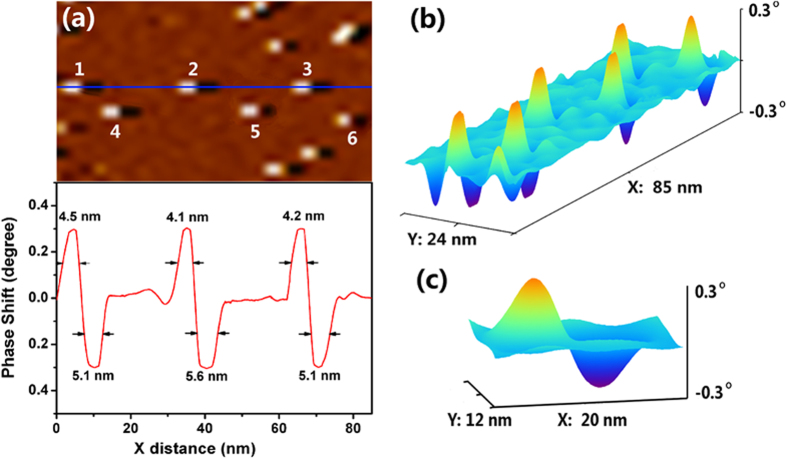
(**a**) Fine magnetic domain structures of individual magnetic nanoparticles and corresponding spectrum line profile; (**b**) corresponding 3-Dimensional (3D) MFM image of indexed six nanoparticles; (**c**) 3D MFM image of particle 6.

The MFM experiment was done in air atmosphere with a commercial MFM (JSPM-5400). A schematic view on the whole measurement procedure is depicted in Fig. 1. The oscillation frequency (ω_c_) of the piezoelectric element was near the resonant frequency of the cantilever (ω_0_). The value of ω_0_ was about 256 kHz and the value of *Q* was about 500. The soft magnetic ferrite core is driven by sinusoidal AC voltages (0 ~ 0.016 V) with a frequency ω_m_ of 100 Hz from a signal generator and AC magnetic fields with amplitude of around 0 ~ 7.2Oe are generated successfully on sample surface. Tapping and lift mode AFM/MFM scans were carried out using a high-coercivity FePt tip, which is perpendicularly magnetized. The tip was coated with a 20 nm FePt film and the radius is 10 nm. The lift height (tip-sample distance) was kept at 10 nm.

## Additional Information

**How to cite this article**: Li, X. *et al.* Quantitatively probing the magnetic behavior of individual nanoparticles by an AC field-modulated magnetic force microscopy. *Sci. Rep.* **6**, 22467; doi: 10.1038/srep22467 (2016).

## Figures and Tables

**Table 1:** Measured phase shift and calculated magnetic moment of the MNPs in Fig. 4(a).

Particle No.	phase shift δ_φ_ (degree)			m_s_ (10^−19^ A m^2^)
	positive	negative	average	
1	0.297	0.298	0.299	2.76
2	0.303	0.305	0.304	2.82
3	0.300	0.299	0.300	2.79
4	0.302	0.298	0.300	2.78
5	0.295	0.302	0.299	2.77
6	0.304	0.286	0.295	2.74
